# The Diagnostic Validity and Reliability of an Internet-Based Clinical Assessment Program for Mental Disorders

**DOI:** 10.2196/jmir.4195

**Published:** 2015-09-21

**Authors:** David Phong Nguyen, Britt Klein, Denny Meyer, David William Austin, Jo-Anne M Abbott

**Affiliations:** ^1^ National eTherapy Centre Swinburne University of Technology Hawthorn, VIC Australia; ^2^ Centre for Biopsychosocial and eHealth Research & Innovation; Collaborative Research Network; and the School of Health Sciences & Psychology Federation University Australia Ballarat, VIC Australia; ^3^ National Institute for Mental Health Research The Australian National University Canberra Australia; ^4^ School of Health Sciences Swinburne University of Technology Hawthorn, VIC Australia; ^5^ School of Psychology Deakin University Burwood, VIC Australia

**Keywords:** Internet, online, mental health, validity, reliability, assessment, diagnosis, screening, anxiety, depression

## Abstract

**Background:**

Internet-based assessment has the potential to assist with the diagnosis of mental health disorders and overcome the barriers associated with traditional services (eg, cost, stigma, distance). Further to existing online screening programs available, there is an opportunity to deliver more comprehensive and accurate diagnostic tools to supplement the assessment and treatment of mental health disorders.

**Objective:**

The aim was to evaluate the diagnostic criterion validity and test-retest reliability of the electronic Psychological Assessment System (e-PASS), an online, self-report, multidisorder, clinical assessment and referral system.

**Methods:**

Participants were 616 adults residing in Australia, recruited online, and representing prospective e-PASS users. Following e-PASS completion, 158 participants underwent a telephone-administered structured clinical interview and 39 participants repeated the e-PASS within 25 days of initial completion.

**Results:**

With structured clinical interview results serving as the gold standard, diagnostic agreement with the e-PASS varied considerably from fair (eg, generalized anxiety disorder: κ=.37) to strong (eg, panic disorder: κ=.62). Although the e-PASS’ sensitivity also varied (0.43-0.86) the specificity was generally high (0.68-1.00). The e-PASS sensitivity generally improved when reducing the e-PASS threshold to a subclinical result. Test-retest reliability ranged from moderate (eg, specific phobia: κ=.54) to substantial (eg, bulimia nervosa: κ=.87).

**Conclusions:**

The e-PASS produces reliable diagnostic results and performs generally well in excluding mental disorders, although at the expense of sensitivity. For screening purposes, the e-PASS subclinical result generally appears better than a clinical result as a diagnostic indicator. Further development and evaluation is needed to support the use of online diagnostic assessment programs for mental disorders.

**Trial Registration:**

Australian and New Zealand Clinical Trials Registry ACTRN121611000704998; http://www.anzctr.org.au/trial_view.aspx?ID=336143 (Archived by WebCite at http://www.webcitation.org/618r3wvOG).

## Introduction

The diagnosis of mental disorders has many important roles in clinical practice, research, and administration (eg, communication, treatment planning and evaluation, decision making, classification, policy development) [1]. However, there are various issues that limit the practice and utility of diagnostic assessment in traditional face-to-face settings [2-5]. For example, clinicians typically favor unstructured interviewing despite being prone to bias and error [4], whereas the more reliable structured interviewing format is often overlooked for being cumbersome and costly to administer in everyday practice [5].

The Internet offers various benefits to assist the assessment of mental disorders [6,7]. Internet-based questionnaires can incorporate complex branching and scoring rules, as well as seamlessly present items and feedback in a standardized manner. The Internet also offers minimal ongoing delivery costs, accessibility across diverse population groups, and efficient data collection. Consumer accessibility is typically better than for traditional face-to-face services because it is usually associated with lower cost and greater convenience. Furthermore, the potential anonymity of online assessment facilitates self-awareness and self-disclosure, potentially enabling more valid outcomes [8].

Given these advantages, numerous and diverse online diagnostic assessment tools have been made available. However, published psychometric properties regarding diagnostic outcomes are only available for a small proportion of these. Furthermore, performance varies widely across these reported programs (eg, [9-12]), probably due to differences in program characteristics and study methodologies. For example, Farvolden et al [9] reported on the validity of the Web-Based Depression and Anxiety Test (WB-DAT), a diagnostic screener for depression and anxiety disorders that functions similarly to a structured diagnostic interview based on *Diagnostic and Statistical Manual of Mental Disorders* (Fourth Edition) (*DSM-IV*) criteria. With a clinician-administered Structured Clinical Interview for *DSM-IV* (SCID-IV) as the gold standard, the WB-DAT displayed a high level of diagnostic accuracy in terms of sensitivity (0.71-0.95) and specificity (0.87-0.97). However, results were limited in that participants were recruited from face-to-face clinical trials and may not have represented typical online consumers of the program. Furthermore, the study involved generally low diagnostic base rates that could have biased classification statistics. Nevertheless, the results for the WB-DAT suggest that an online program can achieve a high level of diagnostic sensitivity and specificity.

More recently, Donker et al [10] evaluated the Web Screening Questionnaire (WSQ), which also diagnostically screens for multiple *DSM-IV* disorders (eg, depression, anxiety, and alcohol-related disorders). Unlike the WB-DAT, the WSQ is very brief, with only 1 to 2 items assigned to each disorder and 15 items in total to promote access and completion [10]. In contrast to Farvolden et al’s study, participants (N=502) were recruited online and subsequently completed the WSQ remotely to better represent potential program usage. Compared against a telephone-based Composite International Diagnostic Interview (CIDI) as the gold standard, a refined version of the WSQ displayed generally high sensitivity (0.72-1.00). However, the WSQ demonstrated relatively poor specificity (0.44-0.77) and low positive predictive values (PPV=0.11-0.51) with many false positives, probably due to the small item set. Hence, although the WSQ may be diagnostically sensitive and quick to complete, it does so at the expense of specificity when contrasted to a more comprehensive program such as the WB-DAT.

Within the psychometric literature of online diagnostic programs, test-retest reliability seems to be an important, yet underinvestigated, type of reliability given that numerous factors (eg, changes in test-taking attitudes and lack of control in test environment) could vary online performance and subsequent results between sittings [13]. Only one known study has examined the test-retest reliability of an online diagnostic assessment tool. In Lin et al’s study, participants comprising Taiwanese visitors to an online mental health website repeated the Internet-based Self-assessment Program for Depression (ISP-D), a 9- to 24-item measure of 3 different depressive presentations [11]. The ISP-D was found to have excellent test-retest reliability within 2 weeks (weighted κ=.80), although performance dropped over longer durations (eg, weighted κ=.45 for 2-4 weeks). Although Lin et al’s results are promising, it is unclear whether they can be generalized to programs targeting other disorders and with different population groups.

Given their practical benefits and psychometric evidence, Internet-based diagnostic assessments have been implemented and trialed in “virtual clinics” as a means of rapid assessment and referral to appropriate online interventions [10,14]. One example is the electronic Psychological Assessment and Screening System (e-PASS), which is the focus of this study. Appearing within the Anxiety Online virtual clinic [14] (now renamed as Mental Health Online [15]), the e-PASS predominantly functions as a diagnostic and referral tool for registered users and is the starting point for accessing online treatment programs [14]. For example, a user identified by the e-PASS as having panic disorder would be recommended to complete an online treatment program for panic disorder [14].

Unlike many other diagnostic assessment programs, the e-PASS aims to produce an accurate diagnostic result by incorporating items reflecting diagnostic criteria and severity. The e-PASS also assesses a considerably wider diagnostic breadth, including 21 *DSM-IV* (Text Revision; *DSM-IV-TR*) disorders, compared with most publically available programs, to help accommodate comorbid and lower prevalence disorders. Another distinct attribute of the e-PASS is that it distinguishes the primary diagnosis (ie, the disorder deemed of greatest severity in a presentation) from any secondary disorders. This feature helps users identify their main mental health issue and prioritize treatment recommendations. Finally, the e-PASS focuses on clinical disorders as well as “subclinical” presentations that represent significant symptoms, but do not meet full criteria and severity of a clinical disorder.

Preliminary evaluation has indicated high diagnostic agreement between the e-PASS and community sources (eg, psychologist, counselor, or medical doctor), although results were based on limited survey data [14]. The e-PASS has also undergone usability testing suggesting it offers distinct benefits and advantages (eg, convenience, anonymity, comprehensiveness) compared to a clinician-administered interview (D Nguyen, unpublished PhD thesis, Victoria: Swinburne University, 2013). In particular, the e-PASS has proven to be highly accessible with more than 22,620 completions between October 2009 and June 2014.

As with any diagnostic assessment tool, it is crucial to formally clarify the psychometric properties of the e-PASS. This need is particularly apparent given the e-PASS’ high usage and explicit role in diagnosis and treatment referral as well as outcome measurement in a “virtual” clinic (eg, [14]). Although psychometric evidence for several online assessment programs exist (eg, [9,10]), their findings are limited in reflecting the potential performance of the e-PASS. For example, the e-PASS differs from previously examined programs in terms of identifying a broader range of disorders (including less common disorders such as bulimia nervosa and body dysmorphic disorder) as well as subclinical diagnostic presentations.

Therefore, this study aimed to examine the diagnostic criterion validity and test-retest reliability of the e-PASS involving prospective users completing the e-PASS under relatively naturalistic conditions. This is the first study known to the authors to evaluate both the criterion validity and test-retest reliability of an online multidisorder diagnostic assessment program. This study is also distinct in examining an online diagnostic program that is central to an internationally available open-access “virtual” clinic for mental health disorders. The findings will help facilitate more informed and appropriate use of the e-PASS and further development of the e-PASS and similar online assessment tools.

## Methods

### Ethical Approval

This study was approved by the Swinburne University Human Research Ethics Committee. The study was conducted as part of a larger trial of the Anxiety Online service, which received trial registration with the Australian New Zealand Clinical Trials Registry (ACTRN12611000704998) [14].

### Recruitment

Recruitment targeted prospective e-PASS users. Visitors to the Anxiety Online website who clicked a link to undertake the e-PASS were presented a brief invitation to this research. Those who declined proceeded with the e-PASS per usual, whereas interested individuals were provided with an online plain language statement and consent form. Inclusion criteria required that individuals be 18 years of age or older and residing within Australia (to allow for appropriate follow-up in the advent of participation issues). All clinical populations were welcome, although individuals experiencing acute distress or risk were encouraged to defer participation in the e-PASS study. Recruitment occurred between November 2009 and June 2011. In all, 29 participants were excluded for residing outside of Australia, leaving 616 in the total sample.

### The e-PASS

The e-PASS is a comprehensive assessment program that, in addition to diagnostic assessment, measures a range of factors including sociodemographic background, suicide and psychosis risk, past and current treatment, and preferred learning style. The diagnostic component of the e-PASS consists of more than 500 items grouped into modules representing 21 *DSM-IV-TR* disorders [16]: major depressive disorder (MDD), anxiety disorders (eg, panic disorder), body dysmorphic disorder (BDD), eating disorders (eg, bulimia nervosa), sleep disorders (eg, primary insomnia), alcohol and substance dependence (eg, cannabis dependence), pathological gambling, and somatization disorder. Programmed branching rules allow users to automatically skip nonrelevant items. As a result, users typically only complete a subset of all diagnostic items.

Following e-PASS completion, users are presented with detailed feedback, including a primary diagnosis (ie, the disorder rated as most severe) and any secondary disorders identified. Diagnostic severity is based on the extent that symptom criteria are met and rating scores of distress and interference associated with reported symptoms. A “clinical” diagnostic result is given when all symptom criteria are met and rated with at least “mild” to “moderate” distress and interference. A “subclinical” result is assigned when some, but not all, symptom criteria are met or when all symptom criteria are met but overall severity is rated as less than “mild”.

Items screening for bipolar disorder and schizophrenia, as well as the potential causal role of a medical condition, substance use, and other notable factors (eg, bereavement in depression symptoms) are also reflected in e-PASS diagnostic feedback (see [14] for a more detailed account).

### The Clinical Interview

The clinical diagnostic results of a clinical interview, conducted over telephone, were considered the “gold standard.” The use of telephone interviewing for assessing mental health disorders has support in the literature [17-19]. Interviewers were either fully or provisionally registered psychologists undertaking postgraduate clinical training and were blind to participants’ e-PASS results. Two interview schedules were predominantly used to reach a diagnosis. All interviews commenced with the administration of the Mini International Neuropsychiatric Interview-Plus (MINI-Plus) structured interview schedule. The MINI-Plus is considered practical, while maintaining high diagnostic reliability and validity with the more cumbersome, but highly regarded, SCID-IV [20]. Participants who endorsed MINI-Plus questions indicating some level of anxiety symptoms were also presented the anxiety disorder modules of the Anxiety Disorders Interview Schedule for *DSM-IV-TR* (ADIS-IV), a “gold standard” semistructured interview with demonstrated reliability [21,22]. Participants who indicated sleep difficulties in response to a screening question were also administered the Insomnia Severity Index, a reliable and valid instrument for identifying clinical insomnia [23].

### Procedure

Participants consented by supplying their name, email address, and details of their general practitioner. Participants then completed the e-PASS, which took a mean 25.0 (SD 5.0) minutes, and received diagnostic feedback as per usual. Between June 2010 and June 2011, all e-PASS participants were sent an email invitation to repeat the e-PASS within 35 days of their initial assessment. Interviewers attempted to call participants within 4 weeks of completing the e-PASS. Due to constraints on the interviewing process (eg, interviewers unavailable), a small minority of the total sample (N=616) were not contacted and, unfortunately, it was not noted who those individuals were. Ultimately, of the 162 participants reached, 158 agreed to interviewing whereas 4 declined due to personal reasons. Interviews were completed a mean of 10.4 (SD 7.0) days after e-PASS and had a mean duration of 48.0 (SD 15.0) minutes.

Interviewers commenced with an introduction then proceeded with administering the MINI-Plus followed by the ADIS-IV and Insomnia Severity Index, where relevant. Interviewers were blind to participants’ e-PASS results. Calls ended with participants being invited to other e-PASS–related research activities (eg, qualitative interviewing and online survey of e-PASS experience) not reported in the present study. Following each clinical interview, interviewers completed an assessment summary form including diagnostic outcomes (the presence/absence of a clinical disorder). Interviewers undertook peer supervision and clinical supervision to discuss any clinical concerns and diagnostic issues (eg, differential diagnoses). A random subset of interviews were recorded for interrater reliability testing.

### Statistical Analysis

The e-PASS’ criterion validity was examined by calculating standard classification statistics including sensitivity, specificity, Cohen’s kappa, PPV and negative predictive values (NPV), with diagnostic results of the clinical interview as the criterion (ie, gold standard). Given that classification statistics can be biased by very low diagnostic base rates, only clinical disorders with greater than 4% prevalence according to the clinical interview are reported. Other studies have also reported classification statistics with similarly low base rates (eg, [9,10]).

Sensitivity reflects the proportion of people with a positive clinical interview diagnosis who also received a positive e-PASS diagnosis (ie, true positives). Specificity represents the proportion of those with a negative clinical interview diagnosis who also received a negative e-PASS diagnosis (ie, true negatives). Sensitivity and specificity range from 0 to 1, with higher values indicating better accuracy. Although there are no commonly recommended thresholds for sensitivity/specificity, a minimum sensitivity and specificity of 0.70 was considered acceptable to reflect the priority of screening accuracy [10].

The PPV is the probability of actually having a disorder given a positive diagnosis by the e-PASS, whereas NPV refers to the probability of not actually having a disorder given a negative diagnosis of the disorder by the e-PASS [24]. For sensitivity, specificity, PPV, and NPV, 95% confidence intervals based on the Wilson interval [25] were calculated. Confidence intervals of these statistics reflect potential variability influenced by diagnostic base rates (ie, wider estimates resulting from lower base rates). It is worth noting that previous studies evaluating similar programs (eg, [9,10]) have not included confidence intervals.

Cohen’s kappa [26] measures diagnostic agreement beyond that expected by chance [27]. Kappa values were interpreted following guidelines proposed by Landis and Koch [28]: .01-.20=slight, .21-.40=fair, .41-.60=moderate, .61-.80=substantial, and .81-1.00=almost perfect agreement.

Kappa was also used to measure diagnostic agreement between initial and repeated e-PASS results. The McNemar test examined whether there were systematic changes in diagnosis from test to retest. A significant result implies the need to reject the null hypothesis that the clinical diagnosis for a particular disorder has remained consistent between test and retest, and an examination of the contingency table can then show whether the inconsistency reflects a pattern of change from a positive to negative or negative to positive diagnosis from test to retest [29].

## Results

### Overview

The total sample comprised of 616 people, 443 (71.9%) female and 173 (28.1%) male, with a mean age of 37.7 (SD 12.9) years. The clinical interview sample comprised of 158 people within the total sample. Table 1 shows the sociodemographic characteristics of the total and clinical interview samples. Chi-square tests found no significant differences between the clinical interview sample and the total sample in relation to these sociodemographic variables. A comparison in treatment access showed that a greater proportion were currently accessing treatment within the clinical interview sample (87/158, 55.1%) than the total sample (290/616, 47.1%), but it was not statistically significant (χ^2^_1_=3.4, *P=*.06). Furthermore, results indicated cognitive behavioral therapy access was significantly more prevalent among the clinical interview sample (n, 21.2%) than the total sample (n, 14.3%; χ^2^_1_=6.0, *P=*.01).

Given so few of the clinical interview subsample (ie, 12 of 158) were eventually recorded, it was decided not to proceed with interrater reliability analysis.

**Table 1:** Demographic variables of total sample and clinical interview subsample.

Sociodemographic and treatment factors		Total sample, n (%) N*=*616	Clinical interview subsample, n (%) n=158	χ^2^ (*df*)	*P*
**Gender**				0.2 (1)	.65
	Male	173 (28.1)	42 (26.6)		
	Female	443 (71.9)	116 (73.4)		
**Relationship**				0.7 (4)	.94
	Married	175 (28.4)	44 (27.8)		
	Single	169 (27.4)	44 (27.8)		
	De facto	172 (28.0)	46 (29.1)		
	Separated or divorced	66 (10.7)	14 (8.9)		
	Other	34 (5.5)	10 (6.3)		
**Country of birth**				3.0 (5)	.70
	Australia	453 (73.5)	117 (74.1)		
	United Kingdom	53 (8.6)	14 (8.9)		
	Asian countries	30 (4.9)	9 (5.7)		
	United States	22 (3.6)	2 (1.3)		
	European country (except UK)	22 (3.6)	6 (3.8)		
	Other	36 (5.8)	10 (6.3)		
**Setting**				2.6 (3)	.45
	Metropolitan	384 (62.3)	104 (65.9)		
	Regional	155 (25.2)	36 (22.8)		
	Rural	65 (10.6)	13 (8.2)		
	Remote	12 (1.9)	5 (3.2)		
**Highest schooling**				3.7 (3)	.29
	Year 9 or less	36 (5.8)	7 (4.4)		
	Year 10	70 (11.4)	11 (7.0)		
	Year 11	41 (6.7)	12 (7.6)		
	Year 12	469 (76.1)	128 (81.0)		
**Highest postsecondary education**				6.0 (5)	.30
	None	89 (14.4)	17 (10.8)		
	Current undergraduate	83 (13.4)	15 (9.5)		
	Undergraduate	144 (23.4)	40 (25.3)		
	Postgraduate	117 (19.0)	38 (24.1)		
	Diploma, apprenticeship, trade	92 (14.9)	22 (13.9)		
	Certificate	91 (14.8)	26 (16.5)		
**Employment**				2.6 (6)	.86
	Full time	235 (38.1)	65 (41.1)		
	Part time	175 (28.4)	42 (26.6)		
	Disability, maternity, sick leave	44 (7.1)	10 (6.3)		
	Home duties/carer	43 (7.0)	8 (5.1)		
	Retired	19 (3.1)	7 (4.4)		
	Unemployed	63 (10.2)	17 (10.8)		
	Other (eg volunteer, student)	37 (6.0)	9 (5.7)		
Receiving current mental health assistance		290 (47.1)	87 (55.1)	3.4 (1)	.06
Current cognitive behavior therapy access		88 (14.3)	33 (20.9)	6.0 (1)	.01

### Diagnostic Validity

Only 10 of the 21 disorders targeted by the e-PASS had sufficient base rates to warrant meaningful classification statistics. Among these, measures of diagnostic accuracy indicated mixed performance (Table 2). Kappa values indicated the e-PASS clinical diagnoses of generalized anxiety disorder (GAD; κ=.37) and obsessive-compulsive disorder (OCD; κ=.39) had fair agreement with the clinical interview. The remaining disorders reflected moderate (bulimia nervosa: κ=.47) to substantial (panic disorder: κ=.62) agreement. Sensitivity ranged from 0.43 (alcohol dependence) to 0.86 (MDD), with half of the disorders falling below the acceptable value of 0.70. When taking into account confidence intervals, sensitivity estimates ranged from as low as 0.16 (OCD, alcohol dependence) to a maximum of 0.94 (MDD). In contrast, specificity varied between 0.68 (GAD) and 1.00 (alcohol dependence), with most values greater than 0.90. Estimated specificity values remained generally greater than 0.70 even after considering confidence intervals.

The PPVs primarily varied between 0.45 (posttraumatic stress disorder; PTSD) and 1.00 (alcohol dependence). The NPVs were consistently higher for most disorders, with the smallest magnitude being 0.80 (social phobia) and the remainder equal to or greater than 0.90. From these predictive values, an e-PASS clinical diagnosis appeared to have a low to moderate likelihood of reflecting a positive clinical diagnosis depending on the disorder, whereas a negative e-PASS diagnosis in general was far more likely to be accurate.

Further analyses examined the extent to which an e-PASS clinical or subclinical diagnostic associated with a clinical interview clinical diagnosis. Again, only 10 disorders were considered because of limited base rates and Table 3 summarizes the resulting classification statistics. When considering both a subclinical and clinical e-PASS result as a positive diagnosis, sensitivity ranged from 0.67 (BDD) to 0.98 (MDD) and equaled or exceeded 0.90 for 5 disorders. Specificity was generally lower and varied between 0.38 (MDD) and 0.89 (bulimia nervosa), with only 5 disorders considered acceptable in terms of exceeding 0.70. Kappa values of the e-PASS subclinical/clinical diagnoses remained significant (*P*<.001) and ranged from .18 (PTSD) to .47 (panic disorder, social phobia), with most considered fair (ie, .20-.40) in diagnostic agreement with a clinical interview clinical diagnosis.

The PPVs were generally smaller than those seen when classification was based on the e-PASS clinical diagnosis alone. Only panic disorder and social phobia maintained moderate PPVs with values of 0.48 and 0.58, respectively. As a result of the lower threshold for a positive e-PASS diagnostic result (ie, subclinical rather than clinical diagnosis), the NPVs accordingly increased for all the disorders, with the majority greater than 0.95. This indicates that an individual with the absence of a relevant clinical disorder is very unlikely to receive a positive e-PASS subclinical or clinical diagnosis for that disorder.

**Table 2:** Classification statistics of e-PASS clinical diagnoses against clinical interview clinical diagnoses (n=158).

e-PASS diagnosis		Clinical interview, n		κ^a^	Sensitivity (95% CI)	Specificity (95% CI)	PPV (95% CI)	NPV (95% CI)
		Yes	No					
**Panic disorder**				.62	0.71 (0.55-0.84)	0.91 (0.85-0.95)	0.69 (0.53-0.82)	0.92 (0.86-0.95)
	Yes	25	11					
	No	10	112					
**GAD**				.37	0.78 (0.62-0.88)	0.68 (0.59-0.76)	0.45 (0.34-0.57)	0.90 (0.82-0.95)
	Yes	31	38					
	No	9	80					
**Social phobia**				.52	0.60 (0.47-0.71)	0.90 (0.84-0.96)	0.77 (0.63-0.87)	0.80 (0.72-0.86)
	Yes	34	10					
	No	23	91					
**PTSD**				.52	0.75 (0.47-0.91)	0.92 (0.87-0.96)	0.45 (0.26-0.66)	0.98 (0.94-0.99)
	Yes	9	11					
	No	3	135					
**OCD**				.39	0.36 (0.16-0.61)	0.97 (0.93-0.99)	0.56 (0.27-0.81)	0.94 (0.89-0.97)
	Yes	5	4					
	No	9	140					
**MDD**				.58	0.86 (0.73-0.94)	0.79 (0.71-0.85)	0.61 (0.46-0.76)	0.94 (0.87-0.97)
	Yes	38	24					
	No	6	90					
**Insomnia**				.53	0.78 (0.62-0.88)	0.82 (0.74-0.88)	0.56 (0.42-0.69)	0.93 (0.86-0.96)
	Yes	28	22					
	No	8	100					
**BDD**				.51	0.67 (0.39-0.86)	0.94 (0.89-0.97)	0.47 (0.26-0.69)	0.97 (0.94-1.00)
	Yes	8	9					
	No	4	137					
**Bulimia nervosa**				.47	0.50 (0.24-0.76)	0.97 (0.92-0.99)	0.50 (0.24-0.76)	0.97 (0.93-0.99)
	Yes	5	5					
	No	5	143					
**Alcohol dependence**				.59	0.43 (0.16-0.75)	1.00 (0.98-1.00)	1.00 (0.44-1.00)	0.97 (0.94-0.99)
	Yes	3	0					
	No	4	151					

^a^ All kappa values *P*<.001.

**Table 3:** Classification statistics of the e-PASS subclinical or clinical diagnoses against clinical interview clinical diagnoses (n=158).

e-PASS diagnosis		Clinical interview, n		κ^a^	Sensitivity (95% CI)	Specificity (95% CI)	PPV (95% CI)	NPV (95% CI)
		Yes	No					
**Panic disorder**				.47	0.89 (0.74-0.95)	0.72 (0.64-0.79)	0.48 (0.36-0.60)	0.96 (0.89-0.98)
	Yes	31	34					
	No	4	89					
**GAD**				.21	0.92 (0.88-0.97)	0.40 (0.31-0.49)	0.34 (0.26-0.44)	0.94 (0.84-0.98)
	Yes	37	71					
	No	3	47					
**Social phobia**				.47	0.86 (0.75-0.93)	0.65 (0.56-0.74)	0.58 (0.48-0.68)	0.89 (0.80-0.94)
	Yes	49	35					
	No	8	66					
**PTSD**				.18	0.92 (0.65-0.99)	0.62 (0.54-0.70)	0.17 (0.01-0.27)	0.99 (0.94-1.00)
	Yes	11	55					
	No	1	91					
**OCD**				.33	0.79 (0.52-0.92)	0.81 (0.73-0.86)	0.28 (0.17-0.44)	0.97 (0.93-0.99)
	Yes	11	28					
	No	3	116					
**MDD**				.24	0.98 (0.88-1.00)	0.38 (0.29-0.47)	0.38 (0.29-0.37)	0.98 (0.88-1.00)
	Yes	43	71					
	No	1	43					
**Insomnia**				.23	0.97 (0.86-1.00)	0.42 (0.33-0.51)	0.33 (0.25-0.42)	0.98 (0.90-1.00)
	Yes	35	71					
	No	1	51					
**BDD**				.35	0.67 (0.39-0.86)	0.88 (0.81-0.92)	0.31 (0.17-0.50)	0.97 (0.92-0.99)
	Yes	8	18					
	No	4	128					
**Bulimia nervosa**				.45	0.90 (0.60-0.98)	0.89 (0.82-0.93)	0.35 (0.19-0.54)	0.99 (0.96-1.00)
	Yes	9	17					
	No	1	131					
**Alcohol dependence**				.26	0.86 (0.49-0.97)	0.83 (0.77-0.89)	0.19 (0.09-0.36)	0.99 (0.96-1.00)
	Yes	6	25					
	No	1	126					

^a^ All kappa values *P*<.001.

### Test-Retest Reliability

Of the 60 participants who repeated the e-PASS, 39 did so within 25 days of initial completion (mean 7.98, SD 6.63) and were included in reliability analyses. Participants received a mean 5.05 (SD 2.83) and 4.70 (SD 2.65) subclinical or clinical diagnoses on their first and second administration, respectively, and the difference was not significant (*t*_38_=1.56, *P*=.13).

Table 4 presents the cross-tabulation of e-PASS clinical diagnoses between initial completion and retesting, as well as the significance level of the McNemar test, the percentage agreement, and the kappa agreement coefficient. Due to the small sample size, the exact binomial probability of the data was used to calculate the McNemar test [30]. This was not significant (*P*>.05) for all disorders considered, indicating a similar likelihood of change from nonclinical to clinical diagnosis and vice versa between testing and retesting results. However, this could also be a result of an underpowered McNemar test given that the sample size was only n=39.

All kappa values were significant and reflected generally strong diagnostic agreement between test and retest. Kappa was particularly high for bulimia nervosa and panic disorder, each of which was associated with more than 90% agreement. There was less agreement for insomnia, MDD, and specific phobia, although kappa values were still considered moderate to substantial. An inspection of cases with disagreement found that most involved a change from a subclinical/clinical to clinical/subclinical (respectively) result. For example, 4 of 5 cases of disagreement for specific phobia included a change from a clinical to subclinical diagnosis, whereas the remaining case was of a change from neither a subclinical or clinical diagnosis to a clinical diagnosis of specific phobia.

**Table 4:** Test-retest reliability of e-PASS clinical diagnoses (n=39).

Test		Retest, n		Agreement, %	*P* ^a^	κ^b^
		Yes	No			
**Panic disorder**				94.9	.50	.83
	Yes	6	2			
	No	0	31			
**Social phobia**				87.1	.50	.71
	Yes	10	2			
	No	3	24			
**GAD**				84.6	.22	.67
	Yes	11	5			
	No	1	22			
**Specific phobia**				87.2	.22	.54
	Yes	4	4			
	No	1	30			
**PTSD**				89.8	.63	.61
	Yes	4	3			
	No	1	31			
**MDD**				78.5	.73	.57
	Yes	11	5			
	No	3	20			
**Bulimia nervosa**				97.4	>.99	.87
	Yes	10	1			
	No	1	27			
**BDD**				84.6	.22	.60
	Yes	7	5			
	No	1	26			
**Insomnia**				77.0	>.99	.53
	Yes	12	4			
	No	5	18			

^a^ McNemar test *P* values.

^b^ All kappa values significant at *P*<.001.

## Discussion

The e-PASS is a free, internationally available, online diagnostic assessment (and referral) program for numerous mental disorders. As with any diagnostic tool, particularly one that is highly accessible and can be independently undertaken, there is a need to ensure the e-PASS is valid and reliable. Hence, this study evaluated the psychometric properties of the e-PASS, focusing on its diagnostic criterion validity and test-retest reliability. To enhance the ecological validity of the study findings, participants were recruited online and represented prospective e-PASS users completing the program under generally naturalistic conditions.

The e-PASS was found to have mixed diagnostic agreement with the semistructured clinical interview (ie, the gold standard), varying from fair (eg, OCD) to substantial (eg, panic disorder) agreement. Compared to previously evaluated programs, the e-PASS’ diagnostic sensitivity generally exceeded some (eg, Internet-administered CIDI-Short Form [12]), but not other programs (eg, WB-DAT [9], WSQ [10]). In contrast, the e-PASS’ specificity was generally high, resulting in far less false-positive results than certain programs (eg, WSQ [10]). Predictive statistics suggest that a positive e-PASS result had at least a 45% probability of accurately reflecting an actual disorder, whereas a negative e-PASS result for most disorders was correct in more than 90% of cases. The latter suggests a general strength of the e-PASS is its ability to rule out a disorder, which could be beneficial in minimizing burden associated with false-positive clinical diagnoses (eg, stigma, unnecessary follow-up assessment, and treatment).

Among previously reported programs, the e-PASS most closely resembles the WB-DAT [9]. When considering mutual disorders, the e-PASS produced similar psychometrics to the WB-DAT, except in the cases of OCD and PTSD, where the e-PASS clinical result was noticeably less sensitive. It is worth remembering that psychometric results of the WB-DAT [9] were based on a sample recruited from a face-to-face clinic population consisting of generally lower diagnostic base rates compared to those seen in this study. Furthermore, the e-PASS assesses a wider range of disorders than the WB-DAT and most other programs. To the best of the authors’ knowledge, this is the first study that has reported the psychometric performance of an online program that identifies BDD and bulimia nervosa.

Although the e-PASS screens particularly well for certain disorders (eg, panic disorder, MDD), it seems lacking for others (eg, OCD) when considering the combination of low sensitivity and diagnostic agreement with the clinical interview. Various factors could help explain these mixed classification statistics (eg, imprecise wording of some e-PASS items or unreliable diagnostic criteria for certain disorders). Given that e-PASS specificity often exceeded sensitivity values, one likely explanation is that the e-PASS’ diagnostic threshold was too high for particular disorders. In support of this, additional analyses found that sensitivity values consistently improved and exceeded 90% for some disorders (while maintaining reasonable specificity) when considering an e-PASS “subclinical” or “clinical” result as predictive of an actual clinical disorder. This suggests that the majority of actual clinical disorder cases at least received an e-PASS diagnosis of subclinical, if not clinical severity, which provides some reassurance in terms of notifying e-PASS users of potential mental health issues. Furthermore, the e-PASS is designed so that a subclinical result also prompts access to associated online treatment programs or recommendations of further assessment (eg, face-to-face consultation with a health professional) for follow-up.

Nevertheless, the results of this study suggest one way of improving the e-PASS’ screening properties in terms of maximizing sensitivity would be to reduce the diagnostic threshold (eg, so that a subclinical result is identified as a clinical disorder). However, this in turn would increase false-positive results, decreasing specificity. The extent to which diagnostic thresholds should be reduced will depend on the impact on the respective sensitivity and specificity properties, determined using receiver operating characteristic (ROC) analyses (D Nguyen, unpublished PhD thesis, Victoria: Swinburne University, 2013). A further consideration is the broader impact of accurate/inaccurate results (eg, potential burden of diagnosis including financial costs, stigma, and access of ineffective treatment) which further contributes to the overall utility of the e-PASS.

The e-PASS also demonstrates strong test-retest reliability for identifying a clinical disorder (particularly for panic disorder and bulimia nervosa) over an average of approximately 1 week and a maximum of 25 days. Compared to the ISP-D online screener for MDD [11], the e-PASS produced comparable consistency in identifying MDD. The results of this study are the first to document the test-retest reliability of an online diagnostic assessment program for the other reported disorders (eg, anxiety disorders, insomnia, bulimia nervosa). In general, the e-PASS’ test-retest reliability measures are comparable to those of a computer-assisted administration of the CIDI [31] and a clinician-administered MINI [32].

In this study, the few e-PASS cases with test-retest discrepancies were just as likely to reflect a diagnostic change from clinical to nonclinical compared with nonclinical to clinical. However, this result may have stemmed from underpowered statistical testing given the smaller than expected sample size. On closer inspection, test-retest discrepancies were generally subtle and tended to involve changes from clinical to subclinical results (and vice versa). This may have reflected actual symptom changes given the instability of certain disorders (eg, MDD) over the retesting period of up to 25 days after initial completion. Unfortunately, the reliability sample was too small to limit the analysis to those with shorter test-retest intervals (eg, 1 week). Overall, e-PASS results appear to be generally stable over the short term, which suggests that the potential variability of the online experience does not pose a significant risk to test-retest reliability.

Several limitations should be considered when interpreting the current findings. Firstly, insufficient clinical interviews were recorded to analyze interrater reliability. Also, the administration order of the e-PASS and clinical interview was not counterbalanced and participants’ viewing of e-PASS results in particular may have biased subsequent interview responses. The period between e-PASS and clinical interview completion (mean approximately 10 days) as well as between test and retest of the e-PASS (mean approximately 8 days) may have led to actual symptom changes in some cases. Therefore, the reported validity and reliability statistics could be conservative estimates. Furthermore, the limited number of participants repeating the e-PASS prompts the need for further reliability testing with a larger sample, while also possibly indicating that the e-PASS has low acceptability to some users. Indeed, separate research (D Nguyen, unpublished PhD thesis, Victoria: Swinburne University, 2013) has suggested that some of the e-PASS users were deterred from further use due to certain factors (eg, length, perceived repetition, lack of immediate assistance and support).

Participant recruitment targeted prospective e-PASS users to enhance the ecological validity of findings. Although not reported in this study, the sociodemographic characteristics (eg, gender, employment and marital status, education level) of the approximately 13,000 individuals who completed the e-PASS between October 2009 and October 2012 largely resemble those of this study sample. Nevertheless, the extent to which results based on this study’s sample can be generalized to all e-PASS users requires a more detailed analysis of participant characteristics as well as their potential relationship with psychometric properties. For example, it may be that certain individual characteristics (eg, education level) could be more conducive for e-PASS diagnostic validity or reliability.

With the introduction of *DSM-5* [33], there is a need to revise the e-PASS in-line with new criteria and reevaluate its psychometric properties. Program changes will be minor for most disorder modules (eg, for MDD), although some will require substantial changes (eg, PTSD). Interestingly, the best performing e-PASS diagnoses (eg, MDD and panic disorder) are also those with relatively little criteria change from *DSM-IV-TR* to *DSM-5*. The e-PASS targets 21 disorders, but many of these (eg, anorexia nervosa, pathological gambling, substance disorders) were not examined due to very low diagnostic base rates in the sample. Therefore, further evaluation could involve specific population groups to clarify the e-PASS psychometric properties for these disorders. Additional psychometric evaluation could also consider properties such as the internal reliability of individual e-PASS items, although this would require a much larger sample size as well as modifications to the e-PASS form (eg, removing branching rules) to provide a suitable dataset for analysis.

New means of online diagnostic screening raises the issue of whether to replace, adapt, or supplement Internet-based programs such as the e-PASS. There is potential, for example, to incorporate audiovisual content (eg, [34]) that could enhance accessibility and acceptability. In light of its mixed diagnostic performance, Internet-based screening could also be followed up with clinician interviewing via videoconferencing (eg, [35,36]) or Web chat (eg, [37,38]). Online assessment could also be complemented with mobile-based applications measuring in-the-moment symptoms via questionnaires [39] or audiovisual cues (eg, speech and body language) of the respondent [40].

In contrast to diagnostic screeners, the use of online clinical scales focusing on dimensional measures may prove to offer greater utility in the assessment of mental health disorders [41]. Such programs extend beyond Internet administrations of standard paper-and-pencil measures and are becoming increasingly sophisticated. For example, Batterham et al [42] proposed a hierarchical system commencing with brief online prescreening (eg, K6) followed by an administration of relevant disorder-specific scales. Computer adaptive testing based on item response theory also shows promise in terms of efficiently screening latent traits underlying mental disorders (eg, [43,44]).

In the meantime, given the utility of a diagnosis in clinical practice [1], there is still arguable value in offering Internet-based questionnaires that produce diagnostic results and directly query diagnostic criteria as similar to the approach of gold standard structured clinical interview schedules [5]. As this study shows, an Internet-based diagnostic assessment program can produce diagnostic results that have high test-retest reliability and, at least for certain disorders, high criterion validity. Despite their potential psychometric limitations, these programs could be incorporated into traditional clinical practice alongside other imperfect assessment means (eg, unstructured interviewing) to broaden assessment information and improve overall diagnostic accuracy [3,5]. For many consumers who are unable or unwilling to access traditional services, Internet-based programs could offer a “good enough” alternative for identifying mental health disorders.

In conclusion, this study suggests that the e-PASS has potential for assisting in the diagnosis of mental health disorders and, in doing so, facilitating access to appropriate interventions among other benefits of identifying mental disorders. Nevertheless, further development and evaluation is needed to clarify the full scope of its clinical utility.

## Abbreviations

ADIS-IV: Anxiety Disorder Interview Schedule-IV
BDD: body dysmorphic disorder
CIDI: Composite International Diagnostic Interview
DSM-IV: Diagnostic and Statistical Manual of Mental Disorders (Fourth Edition)
DSM-IV-TR: Diagnostic and Statistical Manual of Mental Disorders (Fourth Edition, Text Revision)
DSM-5: Diagnostic and Statistical Manual of Mental Disorders (Fifth Edition)
e-PASS: electronic Psychological Assessment Screening System
GAD: generalized anxiety disorder
ISP-D: Internet-based Self-assessment Program for Depression
MDD: major depressive disorder
MINI-Plus: Mini International Neuropsychiatric Interview-Plus
NPV: negative predictive value
OCD: obsessive-compulsive disorder
PPV: positive predictive value
PTSD: posttraumatic stress disorder
SCID-IV: Structured Clinical Interview for DSM-IV
WB-DAT: Web-Based Depression and Anxiety Test
WSQ: Web Screening Questionnaire
